# CDDO and ATRA Instigate Differentiation of IMR32 Human Neuroblastoma Cells

**DOI:** 10.3389/fnmol.2017.00310

**Published:** 2017-09-26

**Authors:** Namrata Chaudhari, Priti Talwar, Christian Lefebvre D'hellencourt, Palaniyandi Ravanan

**Affiliations:** ^1^Apoptosis and Cell Survival Research Lab, Department of Biosciences, School of Biosciences and Technology, VIT University, Vellore, India; ^2^Université de La Réunion, Institut National de la Santé et de la Recherche Médicale, UMR Diabète Athérothombose Thérapies Réunion Océan Indien, Saint-Denis de La Réunion, France

**Keywords:** synthetic triterpenoid, differentiation, peroxisome proliferator activated receptor gamma

## Abstract

Neuroblastoma is the most common solid extra cranial tumor in infants. Improving the clinical outcome of children with aggressive tumors undergoing one of the multiple treatment options has been a major concern. Differentiating neuroblastoma cells holds promise in inducing tumor growth arrest and treating minimal residual disease. In this study, we investigated the effect of partial PPARγ agonist 2-cyano-3,12-dioxooleana-1,9(11)-dien-28-oic acid (CDDO) on human neuroblastoma IMR32 cells. Our results demonstrate that treatment with low concentration of CDDO and particularly in combination with all trans retinoic acid (ATRA) induced neurite outgrowth, increased the percentage of more than two neurites bearing cells, and decreased viability in IMR32 cells. These morphological changes were associated with an increase in expression of bonafide differentiation markers like β3-tubulin and Neuron Specific Enolase (NSE). The differentiation was accompanied by a decrease in the expression of *MYCN* whose amplification is known to contribute to the pathogenesis of neuroblastoma. MYCN is known to negatively regulate NMYC downstream-regulated gene 1 (NDRG1) in neuroblastomas. *MYCN* down-regulation induced by CDDO correlated with increased expression of NDRG1. CDDO decreased Anaplastic Lymphoma Kinase (*ALK*) mRNA expression without affecting its protein level, while ATRA significantly down-regulated ALK. Antagonism of PPARγ receptor by T0070907 meddled with differentiation inducing effects of CDDO as observed by stunted neurite growth, increased viability and decreased expression of differentiation markers. Our findings indicate that IMR32 differentiation induced by CDDO in combination with ATRA enhances, differentiation followed by cell death via cAMP-response-element binding protein (CREB) independent and PPARγ dependent signaling mechanisms.

## Introduction

Cell fate determination and differentiation are processes that are crucial in developmental biology, which assigns cells their functional roles and gives them unique identity. Cell differentiation is an essential process. It is a result of differential gene expression, epigenetic modifications, ceased proliferation, and morphological changes converting the precursor cells to specialized ones committed to a specific lineage (Slack, 2012). The differentiation process is paused in certain cancer cells and it is possible that the process could be resumed using relevant differentiation inducing agents, assisting the cancer cells to acquire a terminally differentiated state (Jimenez and Yunis, 1987). Therapeutic application of differentiation therapy successfully made Acute Promyelocytic Leukemia (APL) a curable disease with the use of all trans retinoic acid (ATRA) to differentiate immature leukemic promyelocytes into mature granulocytes (Warrell et al., 1991). In 1971, Barry Pierce and Carol Wallace had put forward a proof of concept that certain malignant cells can differentiate into benign cells. Evidence from their research on squamous cell carcinoma supports the idea that a progeny of malignant stem cells can differentiate into post-mitotic benign squamous cells incapable of forming a tumor (Pierce and Wallace, 1971).

In the first decade of twentieth century, three pioneers: James Homer Wright, William Pepper, and Robert Grieve Hutchison, contributed in delineating the pathology of neuroblastoma in pediatric patients with distinctive clinical presentations (Rothenberg et al., 2009). Neuroblastoma is an extracranial cancer derived from embryonic precursor cells that form the primitive neural crest. These cells are destined to produce multipotent progenitor cells that respond to the ligands and transcription factors, and initiate a regulated signaling cascade to give rise to the peripheral nervous system, the enteric nervous system, pigment cells, Schwann cells, adrenal medullary cells, and cells of the craniofacial skeleton. This differentiation process is forestalled in neuroblastoma making it a heterogeneous disease with variable clinical behavior and outcome. Several studies have reported the underlying genetic and biological causes for neuroblastoma like gene amplification, activating gene mutations, inactivating gene mutations, ploidy, segmental chromosomal abnormalities, single nucleotide polymorphisms, and epigenetic alterations (Schleiermacher et al., 2014). A phenomenon observed in certain younger children with neuroblastoma is a spontaneous complete regression with or without mild chemotherapy (Brodeur and Bagatell, 2014).

Differentiation therapy could be used in combinatorial treatment by inducing regression and steering the cells to complete the differentiation process. Pharmacological modulation of the differentiation signaling pathways in diseases is currently gaining considerable attention. Consequently, it is vital to find novel differentiation triggering agents that can be used in treatment regimens of diseases having a glitch in the differentiation pathways (Kawamata et al., 2006; Andrew et al., 2012). Drugs that are capable of inducing differentiation to stop tumor growth at their earliest stages could be a superior choice either as an alternative or in addition to the existing cornerstone cancer therapies like radiation, chemotherapy, immunotherapy, and angiogenesis inhibition therapy (Allan and Travis, 2005; El-Kenawi and El-Remessy, 2013; Zhou, 2014).

Triterpenoids are a large family of structures synthesized in plants through the cyclization of squalene. Naturally occurring triterpenoids like oleanolic acid (OA) and ursolic acid (UA) are known to have relatively weak anti-inflammatory and anti-carcinogenic activities. In order to increase these activities, new synthetic derivatives have been created (Honda et al., 1999, 2000). 2-cyano-3,12-dioxooleana-1,9(11)-dien-28-oic acid (CDDO), identified as a partial PPARγ agonist, is an inducer of apoptosis in myeloid leukemia cells, osteosarcoma cells, breast cancer cells, and colon cancer cells (Honda et al., 2000; Ito et al., 2001; Lapillonne et al., 2003; Ikeda et al., 2004; Zhang et al., 2004; Chintharlapalli et al., 2005). CDDO is also found to induce differentiation in human osteosarcoma cells and acute myeloid leukemia cells (AML-HL60), and enhances ATRA induced differentiation in NB4 and MR2 AML cells (Ito et al., 2001; Koschmieder et al., 2007; Tabe et al., 2007). Having knowledge of the versatile behavior of CDDO, we set forth a question “Could CDDO induce differentiation of neuroblastoma as well?” After treating the neuroblastoma cells with CDDO, we expected alterations in the following attributes: morphological changes, up-regulation of markers for differentiation, decrease in MYCN levels and finally activation of essential signaling pathways. It was interesting to find that CDDO as a single agent and in combination with ATRA induced differentiation of MYCN amplified IMR32 cells which to our knowledge is a first report demonstrating the ability of CDDO to induce neuroblastoma differentiation.

## Materials and methods

### Reagents and cell cultures

IMR32, a human neuroblastoma cell line was purchased from NCCS (Pune) and was maintained at 37°C in 5% humidified CO_2_ atmosphere in Dulbecco's Modified Eagle Medium (DMEM, HiMedia). Media was supplemented with 10% fetal bovine serum (FBS, HiMedia), 1X antibiotic-antimycotic (HiMedia) and 1X glutamax (Gibco). CDDO and PPARγ antagonist (T0070907) were purchased from Cayman. ATRA was purchased from Sigma Aldrich. Stocks were prepared in DMSO.

### Cell differentiation and neurite staining

For inducing differentiation, IMR32 cells were seeded in a 6-well plate at a density of 50,000 cells per well and treated with CDDO and ATRA alone and in combination for 5 days and the differentiation inducers were replenished on day 3 by changing the media. Cells were pre-incubated with T0070907 for 2 h prior to addition of CDDO. To observe the cell morphology and neurites, cells were washed with DPBS, prefixed for 2 min in media containing 3.7% formaldehyde and further fixed with DPBS containing 3.7% formaldehyde at −20°C. Permeabilization was done using absolute methanol at −20°C and finally cells were stained with methylene blue solution (0.2% methylene blue in methanol). Cells were imaged on EVOS FLoid Imaging Station (Thermo Fisher Scientific) equipped with a monochrome CCD camera. Neurites were traced using ImageJ software with neuron growth plug-in and represented as average total neurite length (μm). For neurite length analysis, detectable neurites were measured from different focuses and cells without neurites were excluded from analyses. The total number and number of cells bearing more than two neurites were counted in 10 random focuses from independent experiments and reported as a percentage. Control cells received the vehicle alone.

### Cell viability

Cell viability was estimated by measuring cellular ATP levels using CellTiter Glo luminescent assay (Promega) (Hannah et al., 2001). The assay uses luciferase reaction to measure ATP, which is proportional to the emitted luminescence signal. Briefly, 2,000 cells per well were seeded in 96-well plate (white) and treated as mentioned above. On day 5 the CellTiter Glo reagent was added to wells (50 μl), mixed and luminescence was measured using a luminometer (Berthold).

The absolute number of viable cells was counted using trypan blue exclusion method. Briefly, 20,000 cells per well were seeded in 24-well plate and treated with differentiation inducers. At different experimental time points, both adhered and suspended cells were collected and stained with 0.4% trypan blue solution. Cells were counted manually using hemocytometer.

### RNA extraction, reverse transcriptase PCR (RT-PCR), and quantitative real-time PCR (qPCR)

After inducing differentiation, cells were harvested at different time points. Total RNA from (both treated and control wells) the cultured cells were isolated using RNAiso Plus (Takara). RNA quality and quantity were assessed by Nanodrop UV-VIS spectrophotometer (Thermo Fisher Scientific). Two micrograms of total RNA from each sample was reverse transcribed to cDNA using Prime Script RT reagent kit (Takara). Using SYBR Premix Ex Taq (Tli RNase H Plus, Takara), RT-qPCR was performed on Applied Biosystems Step one plus PCR machine. The RNA 18S gene was amplified as an internal standard reference gene (invariant control). Fold changes in the target gene expression were normalized to 18S ribosomal RNA gene expression using comparative CT method (2^−ΔΔCT^ method) (Schmittgen and Livak, 2008).

### Immunofluorescence

For immunofluorescence, 50,000 cells were seeded on gelatin coated coverslips in complete media and on the next day were treated with differentiation inducers. On day 5, cells were washed with DPBS, prefixed for 2 min in media containing 3.7% formaldehyde and further fixed with DPBS containing 3.7% formaldehyde at −20°C. Permeabilization was done using absolute methanol at −20°C. Primary antibodies employed for immunocytochemistry are anti-β3-Tubulin (5568, Cell Signaling Technology, Danvers, MA, USA) and anti-Neuron Specific Enolase (NSE) (A3118; ABclonal Technology, USA). Alexa Fluor® 594 Conjugate goat anti rabbit (8889S, Cell Signaling Technology) was used as secondary antibody. Every step was followed according to manufacturer's instructions. The cells were counterstained with DAPI (HiMedia) in phosphate buffered saline for 5 min and the coverslips were mounted using fluoroshield (Sigma Aldrich, St Louis, MO, USA). Fluorescent images were acquired on EVOS FLoid Imaging Station using 20X fluorite objective and LED light cubes containing hard coated filters (blue and red), (Thermo Fisher Scientific). Imaging parameters like brightness and contrast were kept constant throughout the experimental conditions for particular protein expression. No image modifications were done post imaging.

### Western blot analysis

Media was aspirated and cells were washed with DPBS and lysates were obtained using RIPA buffer (20 mM Tris pH 7.5, 150 mM NaCl, 1 mM Na_2_EDTA, 1 mM EGTA, 1% NP-40, 1% Sodium deoxycholate) supplemented with 1 mM PMSF, Protease inhibitor cocktail (Sigma Aldrich) and Phosphatase inhibitor cocktail (PhosSTOP, Roche). Cell lysates were passed several times through insulin syringe and incubated on ice for 30 min. Total protein was estimated by Lowry's method (Lowry et al., 1951). The extracted proteins were diluted and mixed with SDS-PAGE gel loading buffer to achieve a final concentration of 50 μg/20 μl (23 μl aliquots were made for each protein and stored). The first gel run was performed with actin to confirm equal loading. Further to check protein expression, each time a gel was run with 20 μl (50 μg) loading volume. Stripping and re-probing were never performed. However, in a case of significant difference in the molecular weight of two proteins the membrane was cut horizontally. An equal amount of extracted total protein (50 μg) was resolved in 10–12% SDS-PAGE and transferred onto polyvinylidene difluoride difluoride (PVDF) membranes (Fluoro Trans W, Pall Corporation). Blots were blocked with 5% skim milk in Tris buffered saline-Tween 20 (TBST) for 1 h and immunoblotted with specific rabbit anti human antibodies: PPARγ Antibody (2443S) (Cell Signaling Technology); NDRG1 antibody (NBP1-32074) and ALK Antibody (NBP1-00710) (Novus Biologicals); ERK1/2 antibody (A0229), phospho ERK1/2 antibody (AP0472), CREB1 antibody (AP0019), and phospho CREB1 (AP0019) (ABclonal Techology, USA) for 3 h at room temperature. The membrane was then incubated for 1 h with anti-rabbit HRP conjugated secondary antibody (7074, Cell Signaling Technology). Immunocomplexes were visualized using enhanced chemiluminescence (ECL) chemistry (Mruk and Cheng, 2011) and developed on film (Fuji). Equal loading and transfer were validated by probing the membranes with beta actin antibody (8457S, Cell Signaling Technology).

### Statistical analysis

Statistical tests were performed using Graph Pad Prism6 software. Methylene blue staining and immunocytochemistry has been performed several times and reported as a representative image. All other experiments have been done twice and data is represented as an average of two independent experiments performed in triplicates except for trypan blue viability assay that was performed twice in duplicates. One-way ANOVA followed by Tukey's test was applied to compare more than two groups. Two-way ANOVA followed by Tukey's *post-hoc* test was applied to compare multiple groups (two parameters). Figure legends include mean ± SEM and p values for the statistical analysis performed.

## Results

### CDDO alone and in combination with ATRA induces neurite outgrowth and decreases viability in IMR32 cells

**Figure 1:**
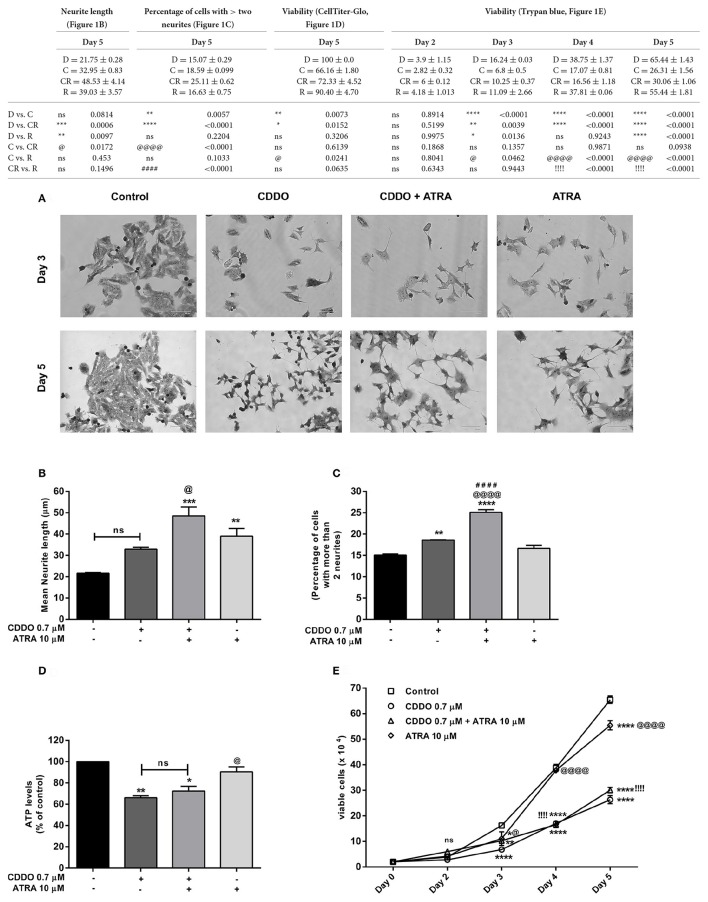
CDDO induces differentiation, enhances ATRA induced differentiation and decreases viability in IMR32 cells. **(A)** Cells were viewed under phase contrast microscope to study the morphological features of methylene blue stained IMR32 cells following treatment with CDDO 0.7 μM and ATRA 10 μM alone and in combination for 5 days. ATRA 10 μM was used as positive control. The images were captured on a microscope equipped with a monochrome camera. **(B)** Stained images in triplicates per treatment condition were opened in ImageJ software and were analyzed used Neuron growth plug-in. Neurites were semi-automatically traced and lengths were expressed as mean neurite lengths. Error bars represent mean ± SEM calculated using one-way ANOVA and Tukey's multiple comparison test. **(C)** The number of cells in treated and non-treated conditions with more than two neurites were counted from random focuses and represented as a percentage. Error bars represent mean ± SEM calculated using one-way ANOVA and Tukey's multiple comparison test. **(D)** Cell viability of IMR32 cells was analyzed using CellTiter-Glo luminescent cell viability assay. Cells were treated with indicated concentrations for 5 days. Error bars represent mean ± SEM calculated using one-way ANOVA and Tukey's multiple comparison test. **(E)** Cell viability of IMR32 cells was alternatively studied using trypan blue dye. Viable cells were counted manually using hemocytometer on day 2, 3, 4, and 5. Two independent experiments were performed in duplicates. Numbers on the y axis represent the total number of viable cells. Error bars represent mean ± SEM calculated using two-way ANOVA and Tukey's multiple comparison test. “_*_” denotes significance with respect to control cells (DMSO treated), “@” denotes significance with respect to CDDO 0.7 μM, and “!/#” denotes significance with respect to ATRA 10 μM. DMSO (D); CDDO (C); CDDO + ATRA (CR); ATRA (R); four indicators: *p* < 0.0001; three indicators: *p* ≤ 0.001; two indicators: *p* ≤ 0.01; one indicator: *p* ≤ 0.05.

To test our hypothesis that synthetic triterpenoid CDDO could induce neuroblastoma differentiation; we initially performed a dose response experiment by treating IMR32 cells with various concentrations of CDDO (0.2, 0.5, 0.7, 1, 1.5, and 2 μM) and observed for neurite outgrowth. We observed neurite outgrowth in treated cells at 0.5 and 0.7 μM of CDDO whereas slightly higher concentrations of (1–2 μM) induced cell death without displaying neuritogenesis in IMR32 cells. Similarly, a dose response experiment was performed with various concentrations of a known differentiation inducer, i.e., ATRA (5, 7.5, 10, and 15 μM) and it revealed that ATRA 10 and 15 μM induced neurite outgrowth. Subsequently, IMR32 cells were treated with 0.7 μM CDDO individually, and also in combination with 10 μM ATRA for 5 days to check the combinatorial effect. For comparison, we limited the ATRA treatment period to 5 days. CDDO treatment as a single agent exhibited delayed morphological changes. However, in combination with ATRA, initial sprouting of neurite was observed as early as day 3 and eventually, a branched neurite network between cells became apparent on day 5 for both the treatment conditions. A comparatively high rate of differentiation was observed in the cells that received the CDDO and ATRA treatment in combination compared to the cells that were treated only with CDDO. Vehicle control cells failed to exhibit aforementioned morphological features and continued to proliferate. At first, methylene blue staining was performed to determine the differentiation features (Figure 1A).

Neurite lengths were then semi-automatically traced on methylene blue stained images using ImageJ software (National Institute of Mental Health, Bethesda, Maryland, USA) with neuron growth plug-in (Universidad Nacionalg Autónoma de México, UNAM) (Fanti et al., 2008; Schneider et al., 2012). Data is represented as average total neurite length. Consistent with the morphological appearance, the cells treated with combination displayed maximum average total neurite length (Figure 1B, Supplementary Figure 1). CDDO treated cells exhibited a slight but significant concentration dependent increase in neurite length with the higher concentration of 1 μM reducing cell viability and neurite outgrowth. A moderate yet significant increase in neurite length was noticed after treatment with CDDO in combination with ATRA, at various combinations of concentrations (Supplementary Figure 2A). The combination treatment also displayed a higher percentage of cells bearing more than two neurites when compared to individual treatments and control cells (Figure 1C).

Treatment with CDDO in both cases had a significant effect on cell viability with respect to untreated cells. CDDO alone and in combination with ATRA significantly reduced cell viability after 5 days of treatment, a phenomenon common to cells undergoing differentiation (Figures 1D,E). Further rounds of treatment up to 10 days with CDDO alone and in combination with ATRA reduced the viability, whereas ATRA treatment exhibited increased proliferation (Supplementary Figure 3). CDDO at slightly higher concentrations (12 μM) induced neurite outgrowth in Neuro2a, mouse neuroblastoma cells (Supplementary Figure 4). However, CDDO failed to induce neurite outgrowth in SHSY5Y, another human neuroblastoma cell line (Supplementary Figure 5).

Additionally, we performed experiments with serum free media, and media containing low percentage serum (0.5%) to check the effect of serum mitogens on IMR32 differentiation. In the absence of serum mitogens, control cells, CDDO, and ATRA treated cells lost the viability in 48 h. In case of medium containing 0.5% serum, CDDO treated cells struggled to produce neurites and maximum cells morphologically appeared dead after 5 days. ATRA induced certain neurite formation in low serum conditions but not as effective as in 10% serum containing media. In addition, reduced proliferation and unhealthy morphology were observed in treated as well as non-treated conditions in low and no serum conditions (Supplementary Figures 6, 7). Altogether, these results suggest that after 5 days of treatment, combination of CDDO with ATRA significantly decreased the cell viability with enhanced induction of neurite outgrowth in IMR32 cells.

### CDDO alone and in combination with ATRA up-regulates differentiation markers

**Figure 2:**
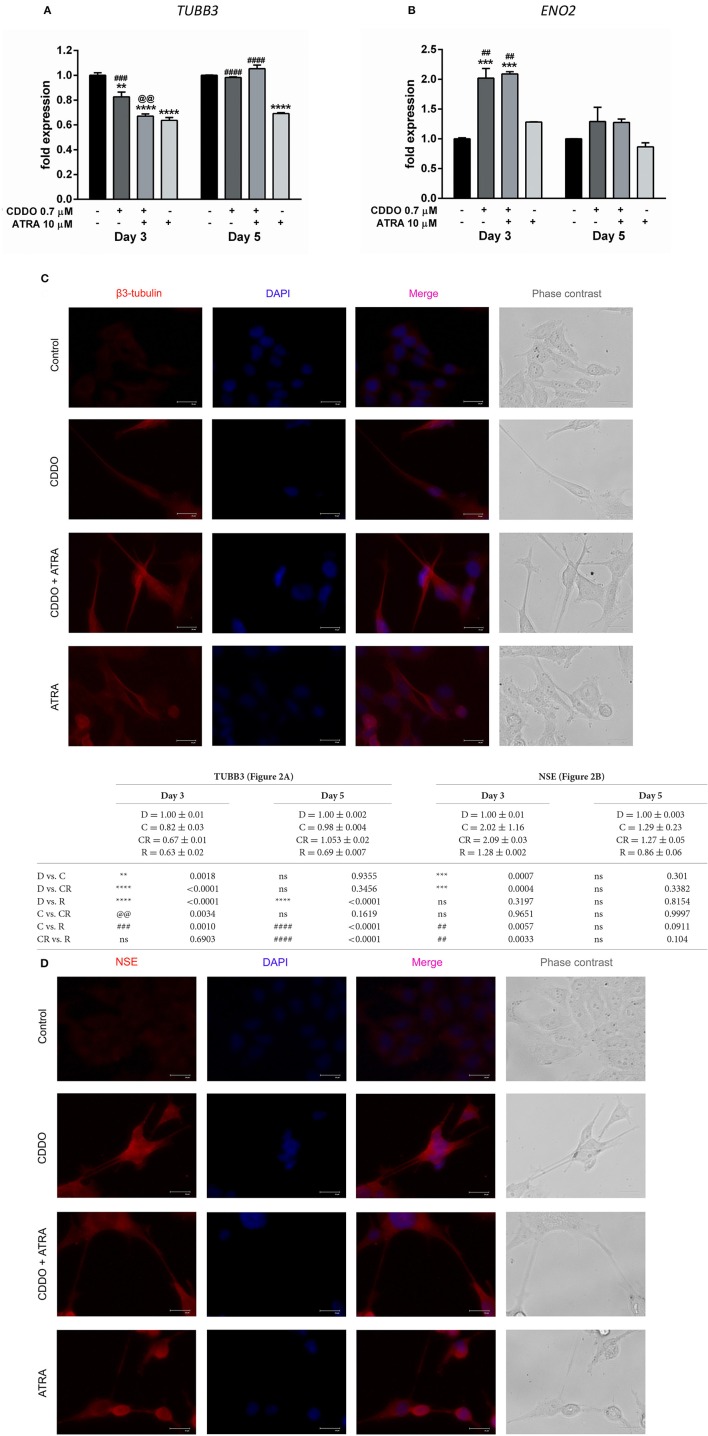
CDDO alone and in combination with ATRA regulates expression of differentiation markers. RT-qPCR analysis was performed for TUBB3 **(A)** and NSE **(B)** mRNA expression in IMR32 cells treated with CDDO 0.7 μM, ATRA 10 μM alone, and in combination for 3 and 5 days. The mRNA expressions were normalized to invariant control 18S. Error bars represent mean ± SEM calculated using two-way ANOVA with Tukey's *post-hoc* test. Representative fluorescent microscopy image showing localization of differentiation markers β3-tubulin **(C)** and NSE **(D)** in IMR32 cells treated with CDDO 0.7 μM, ATRA 10 μM, and in combination for 5 days. Cellular morphology was observed in phase contrast images; protein markers were immunostained red and nuclei were stained blue (DAPI). Co-localization appears pink. “_*_” denotes significance with respect to control cells (DMSO treated), “@” denotes significance with respect to CDDO 0.7 μM and “#” denotes significance with respect to ATRA 10 μM. DMSO (D); CDDO (C); CDDO + ATRA (CR); ATRA (R); four indicators: *p* < 0.0001; three indicators: *p* ≤ 0.001; two indicators: *p* ≤ 0.01; one indicator: *p* ≤ 0.05.

To validate the association of neuronal morphology with the expression of neuron specific markers, we first studied the expression of distinct neuronal markers like β3-tubulin (encoded by *TUBB3*) and NSE (encoded by *ENO2*) by RT-qPCR. Although not exclusive, the expression of β3-tubulin, a neuron associated tubulin isotype and NSE, a neuronal glycolytic isoenzyme is considered to be regulated during neuronal differentiation (Draberova et al., 1998; Jung et al., 2013). In agreement with the observed significant morphological differentiation; the gene expression of NSE was concurrently elevated under the treatment conditions with CDDO. Interestingly, β3-tubulin mRNA expression decreased on day 3 and was maintained on day 5. In contrast, ATRA did not regulate NSE gene expression up to day 5 and decreased β3-tubulin mRNA expression at both time points (Figures 2A,B).

Additionally, immunofluorescence was performed using β3-tubulin and NSE antibodies following the treatment (Figures 2C,D, Supplementary Figures 8, 9). Immunofluorescence results indicated their increased expression in differentiated cells. Also, along with the experimental conditions negative antibody control (antibody diluent buffer) was included to ensure the fluorescence obtained is due to the primary antibody (Supplementary Figure 10). The β3-tubulin expression was majorly extra nuclear in the neurites of cells treated with CDDO and/or ATRA. Pale cytoplasmic immunolabelling was observed in control cells. In the case of NSE, faint nuclear, and significant dispersed staining was observed in the neurites of the cells treated with CDDO, ATRA, and in combination compared to control cells. Altogether, these findings bring to light the potential of CDDO to stimulate differentiation in IMR32 cells in combination with ATRA, as evidenced by an increase in mRNA and protein levels of neuroblastoma differentiation markers.

### CDDO and ATRA alter expression of genes involved in neuroblastoma progression

**Figure 3:**
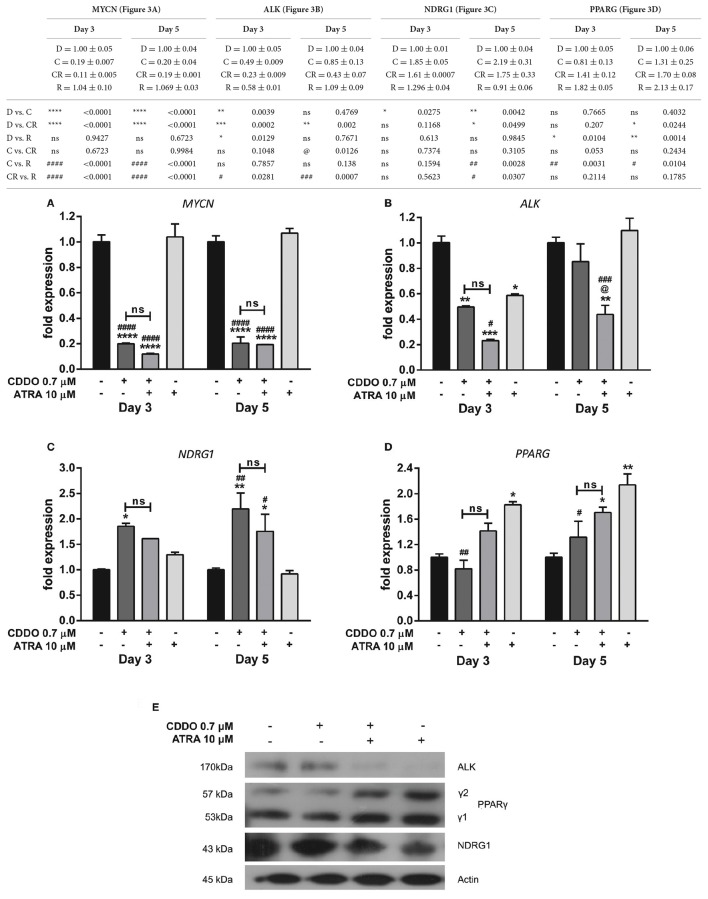
CDDO alone and in combination regulates expression of genes involved in IMR32 differentiation. RT-qPCR analysis was performed at day 3 and day 5 to quantify abundance of indicated mRNA transcripts **(A–D)** in IMR32 cells following treatment with CDDO 0.7 μM and ATRA 10 μM alone and in combination. The mRNA expressions were normalized to invariant control 18S. Error bars represent mean ± SEM calculated using two-way ANOVA with Tukey's *post-hoc* test. **(E)** A representative immunoblot of indicated proteins of IMR32 cells treated with CDDO 0.7 μM and ATRA 10 μM alone and in combination for 5 days (*n* = 2). “_*_” denotes significance with respect to control cells (DMSO treated), “@” denotes significance with respect to CDDO 0.7 μM and “#” denotes significance with respect to ATRA 10 μM. DMSO (D); CDDO (C); CDDO + ATRA (CR); ATRA (R); four indicators: *p* < 0.0001; three indicators: *p* ≤ 0.001; two indicators: *p* ≤ 0.01; one indicator: *p* ≤ 0.05.

A v-Myc related cellular sequence distinct from classical c-Myc oncogene was identified as N-Myc from the homogeneously staining regions (HSR) in IMR32 cells. MYCN was observed to be amplified by 50-folds in IMR32 cells relative to normal placental DNA (Kohl et al., 1983; Schwab et al., 1983). IMR32 cells are therefore MYCN amplified, and to assess the effects of CDDO on IMR32 cell differentiation pertaining to certain key genes regulated in neuroblastoma, RT-qPCR and western blotting was performed. We observed a radical decrease in *MYCN* levels in CDDO treated cells compared to ATRA treated and vehicle control cells. ATRA treatment had no effect on *MYCN* expression (Figure 3A). To verify and associate the *MYCN* expression pattern, MYCN downstream-regulated gene 1 (NDRG1) expression was studied. NDRG1 is repressed in cell lines with MYCN amplification and this down-regulation is known to be associated with MYCN effects in neuroblastomas (Li and Kretzner, 2003). The observed decrease in *MYCN* mRNA levels was accompanied by up-regulation of *NDRG1* mRNA and its protein levels in CDDO treated cells and these results were consistent with IMR32 differentiation. NDRG1 was considerably less expressed in ATRA treated cells, possibly due to high *MYCN* mRNA levels (Figures 3B,E).

Human Anaplastic Lymphoma Kinase (ALK) is a tyrosine kinase receptor and a MYCN targeted oncogene. ALK can be mutated or amplified in neuroblastomas and contributes to the neuroblastomas progression. Also, ALK inhibition has been shown to decrease the biological effects of MYCN amplified cells/tumor growth in xenograft models (Hasan et al., 2013). A gain of function in ALK was observed in high-ALK expressing cells, having either a wild type or mutated receptor (Passoni et al., 2009). IMR32 cells express a wild type ALK receptor with no mutations nevertheless, co-amplification of MYCN and exon 3 and 4 of ALK as a fused single amplicon, has been reported to occur in these cells (Fransson et al., 2015). A considerable reduction in *ALK* mRNA level was observed in CDDO treated cells; whereas ATRA did not regulate *ALK* mRNA level. However, its protein expression was completely inhibited in ATRA treated cells with no change in CDDO treated cells (Figures 3C,E). Because CDDO is a partial PPARγ agonist, we studied if CDDO regulates *PPARG* levels. *PPARG* was found to be regulated by ATRA but not by CDDO. The mRNA levels were mirrored at protein levels as well (Figures 3D,E). These results indicate that CDDO and ATRA together had a distinctive contribution in tailoring the expression profile at both transcriptional and translational levels, reprogramming the cells to complete differentiation by decreasing their oncogenic properties.

### PPARγ antagonist inhibits CDDO induced differentiation in IMR32 cells

**Figure 4:**
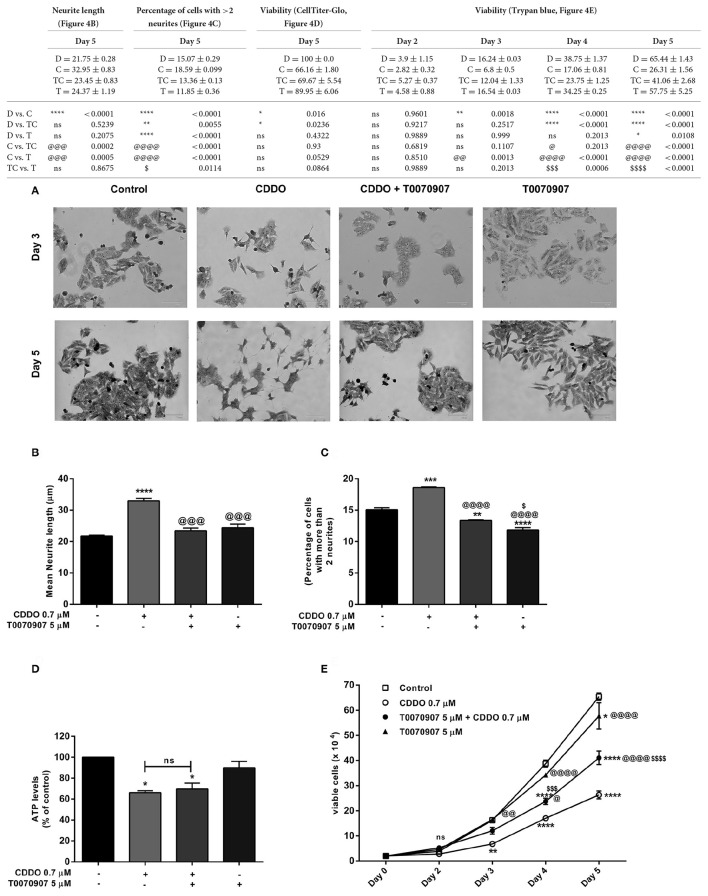
PPARγ antagonist T0070907 inhibits CDDO induced neurite outgrowth and restores viability. Cells were pretreated with T0070907 5 μM for 2 h followed by culture in presence of CDDO 0.7 μM for 5 days and compared to cells treated with CDDO 0.7 μM alone and control cells. **(A)** Cells were viewed under phase contrast microscope to study the morphological features of methylene blue stained IMR32 cells following treatment with CDDO 0.7 μM. The images were captured on a microscope equipped with a monochrome camera. **(B)** Stained images in triplicates per treatment condition were opened in ImageJ software and were analyzed using Neuron growth plug-in. Neurites were semi-automatically traced and lengths were expressed as mean neurite lengths. Error bars represent mean ± SEM calculated using one-way ANOVA and Tukey's multiple comparison test. **(C)** The number of cells in treated and non-treated conditions with more than two neurites were counted from random focuses and represented as a percentage. Error bars represent mean ± SEM calculated using one-way ANOVA and Tukey's multiple comparison test. **(D)** Cell viability of IMR32 cells was analyzed using CellTiter-Glo luminescent cell viability assay. Cells were treated with indicated concentrations for 5 days. Data is represented as an average of two independent experiment performed in triplicates. Error bars represent mean ± SEM calculated using one-way ANOVA and Tukey's multiple comparison test. **(E)** Cell viability of IMR32 cells was analyzed using trypan blue dye. Viable cells were counted manually using hemocytometer on day 2, 3, 4, and 5. Two independent experiments were performed in duplicates. Numbers on the y axis represent the total number of viable cells. Error bars represent mean ± SEM calculated using two-way ANOVA and Tukey's multiple comparison test. “_*_” denotes significance with respect to control cells (DMSO treated), “@” denotes significance with respect to CDDO 0.7 μM and “$” denotes significance with respect to T0070907 5 μM. DMSO (D); CDDO (C); T0070907 + CDDO (TC); T0070907 (T); four indicators: *p* < 0.0001; three indicators: *p* ≤ 0.001; two indicators: *p* ≤ 0.01; one indicator: *p* ≤ 0.05.

**Figure 5:**
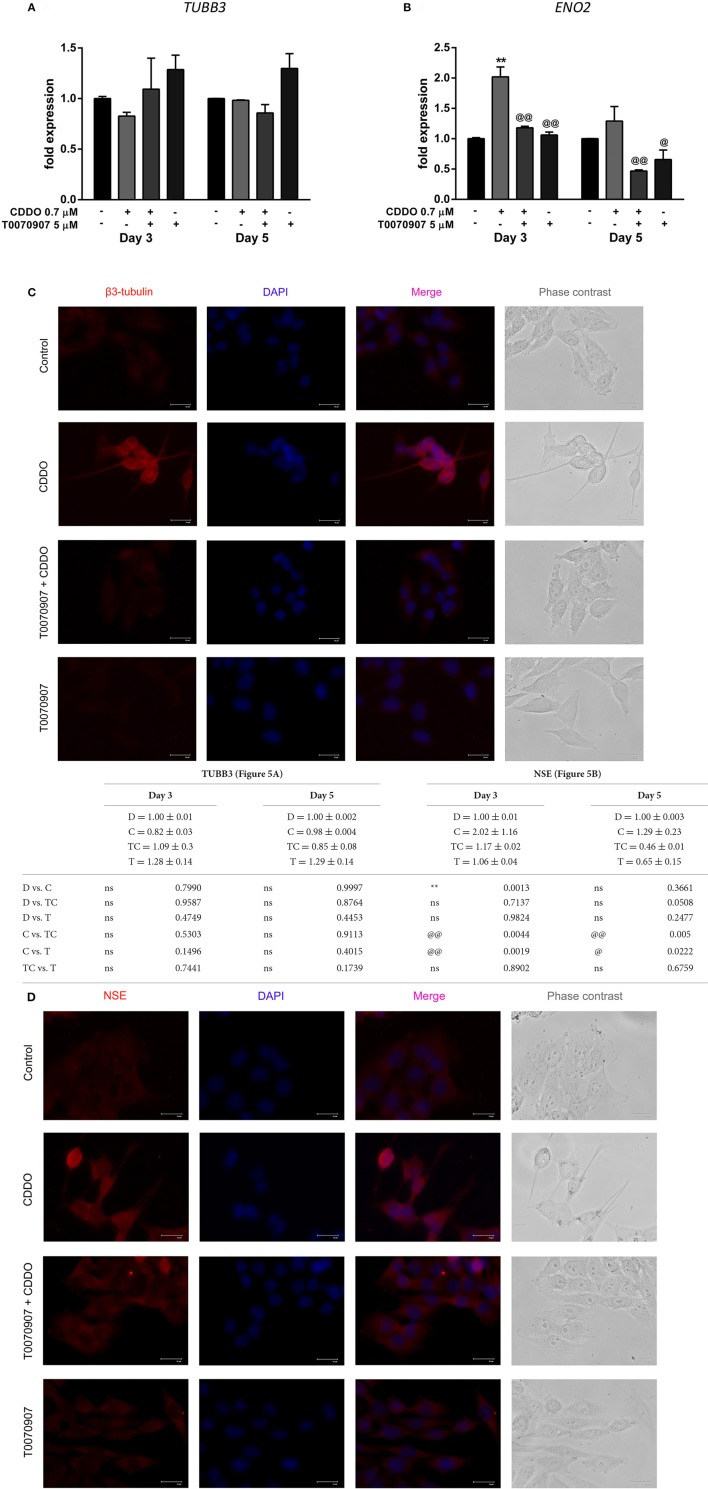
Effect of PPARγ antagonist T0070907 on CDDO regulated genes involved in IMR32 differentiation. Cells were pretreated with T0070907 5 μM for 2 h, followed by culture in presence of CDDO 0.7 μM for 5 days, and compared to cells treated with CDDO 0.7 μM alone and control cells. RT-qPCR analysis was performed for TUBB3 **(A)** and NSE **(B)** mRNA expression in IMR32 cells at day 3 and day 5. The mRNA expressions were normalized to invariant control 18S. Error bars represent mean ± SEM calculated using two-way ANOVA and Tukey's multiple comparison test. Representative fluorescent microscopy image showing localization of differentiation markers β3-tubulin **(C)** and NSE **(D)** in IMR32 cells treated with T0070907 5 μM and CDDO 0.7 μM, for 5 days. Cellular morphology was observed in phase contrast images, protein markers were immunostained red and nuclei were stained blue (DAPI). Co-localization appears pink. “_*_” denotes significance with respect to control cells (DMSO treated) and “@” denotes significance with respect to CDDO 0.7 μM. DMSO (D); CDDO (C); T0070907 + CDDO (TC); T0070907 (T); four indicators: *p* < 0.0001; three indicators: *p* ≤ 0.001; two indicators: *p* ≤ 0.01; one indicator: *p* ≤ 0.05.

Based on these findings, we decided to investigate the response of IMR32 cells to selective PPARγ antagonist T0070907, knowing that CDDO is a partial agonist of PPARγ (Honda et al., 2000). The exposure of 5 μM T0070907 did not display cytotoxicity. The cells were exposed exclusively to T0070907 for 2 h and subsequently cultured for 5 days in presence of differentiation inducers; with the antagonist and inducers being replenished on day 3. The effects of CDDO were considerably inhibited by the antagonist, suggesting that the cellular responses were mediated at least in part by active PPARγ receptor signaling. Antagonism of PPARγ receptor by T0070907 blocked neurite growth promoting effects of CDDO as witnessed by the reduced neurite outgrowth and lengths (Figures 4A,B). Also, pretreatment with T0070907 significantly reduced the percentage of cells bearing more than two neurites compared to that of CDDO treated cells (Figure 4C). T0070907 inhibited CDDO induced neurite outgrowth at a various combination of concentrations (Supplementary Figure 2B). Pretreatment with T0070907 restored the CDDO induced loss in cell viability (Figures 4D,E). Although T0070907 inhibited CDDO induced morphological changes, the *TUBB3* mRNA levels were unaltered by antagonist pretreatment; however, the immunofluorescence analysis displayed reduced expression of β3-tubulin compared to that of CDDO treated cells (Figures 5A,C, Supplementary Figures 8, 9). As shown in Figures 5B,D; clear down-regulation of the differentiation marker NSE was observed after pretreatment with an antagonist at both RNA and protein level. Collectively, T0070907 certainly inhibited neurite outgrowth, reduced the percentage of cells bearing more than two neurites, and decreased the expression of differentiation markers indicating that PPARγ receptor signaling is implicated in reprogramming IMR32 cells to overcome differentiation block.

**Figure 6:**
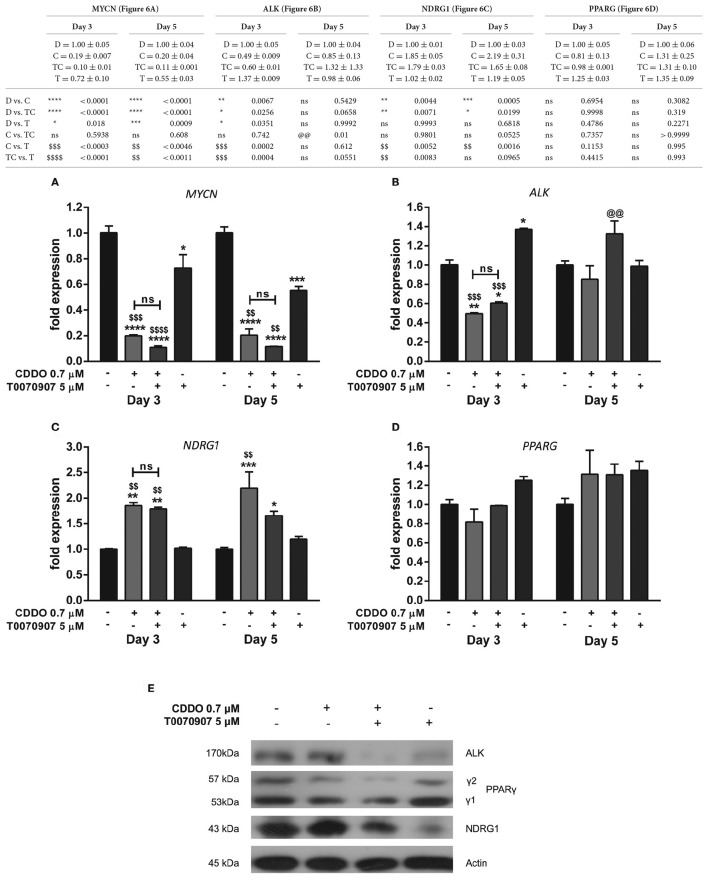
Effect of PPARγ antagonist T0070907 on genes involved in IMR32 differentiation. RT-qPCR analysis was performed at day 3 and day 5 to quantify abundance of indicated mRNA transcripts **(A–D)** in IMR32 cells after pretreatment with T0070907 5 μM for 2 h followed by treatment with CDDO 0.7 μM and compared to cells treated with CDDO 0.7 μM alone and control cells. The mRNA expressions were normalized to invariant control 18S. Error bars represent mean ± SEM calculated using two-way ANOVA and Tukey's multiple comparison test. **(E)** A representative immunoblot of indicated proteins in IMR32 cells pretreated with T0070907 5 μM for 2 h followed by treatment with CDDO 0.7 μM for 5 days (*n* = 2). “_*_” denotes significance with respect to control cells (DMSO treated), “@” denotes significance with respect to CDDO 0.7 μM and “$” denotes significance with respect to T0070907 5 μM. DMSO (D); CDDO (C); T0070907 + CDDO (TC); T0070907 (T); four indicators: *p* < 0.0001; three indicators: *p* ≤ 0.001; two indicators: *p* ≤ 0.01; one indicator: *p* ≤ 0.05.

The antagonist was neither found to restore MYCN mRNA levels that were reduced by CDDO nor to have any contribution in the regulation of PPARG mRNA levels (Figures 6A,D). However, it reversed CDDO regulated *NDRG1* and *ALK* mRNA expression to some extent (Figures 6B,C). Immunoblotting results indicate that active PPARγ signaling was required to maintain ALK expression, as treatment with antagonist inhibited its protein level. CDDO clearly did not regulate ALK protein expression while T0070907 caused a substantial decrease in it. T0070907 exerted a slight inhibition over CDDO induced PPARγ-2 protein level. T0070907 effectively reduced the CDDO induced NDRG1 expression, implying that PPARγ signaling plays an important role in NDRG1 regulation. Moreover, treatment with antagonist alone had reduced NDRG1 expression (Figure 6E). Collectively, antagonist reversed CDDO induced signaling events in IMR32 cells with respect to ALK, NDRG1, and PPARγ-2 protein level signifying that PPARγ signaling is involved in regulating their levels.

### Signaling pathways involved in CDDO and ATRA induced IMR32 differentiation

**Figure 7:**
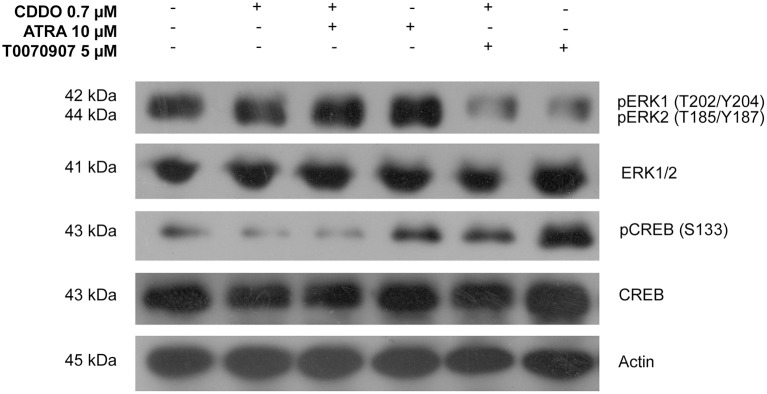
Signaling pathways involved in IMR32 differentiation. A representative immunoblot of indicated proteins in IMR32 cells pretreated with T0070907 5 μM for 2 h followed by treatment with CDDO 0.7 μM, ATRA 10 μM, and in combination for 72 h (*n* = 2).

ERK1/2 and cAMP-response-element binding protein (CREB) activation was studied to identify the signaling mechanisms independent of receptor binding. CREB phosphorylation at Ser133 is considered important for the binding of its co-activator CREB binding protein (CBP) to form a transcriptionally active complex (Gonzalez and Montminy, 1989). Among the several identified kinases that activate CREB, we studied the phosphorylated ERK1/2 levels during IMR32 differentiation. CREB is not a direct downstream target of ERK1/2, instead it is phosphorylated by ERK1/2 activated 90 kDa Ribosomal Protein S6 Kinases (RSKs) (Xing et al., 1996). Treatment with ATRA 10 μM for 72 h resulted in an increased ERK1/2 activation and phosphorylation of CREB. ATRA induced CREB activation was inhibited by co-treatment with CDDO 0.7 μM. CDDO was found to decrease CREB phosphorylation without ERK1/2 activation. However, antagonizing PPARγ signaling displayed increased levels of phosphorylated CREB without ERK1/2 activation. CDDO inhibited CREB activation, could perhaps be a PPARγ dependent mechanism, as antagonist treatment resulted in an increased CREB phosphorylation (Figure 7). In summary, ATRA clearly activated ERK1/2-CREB signaling, whereas CDDO inhibited CREB activation via active PPARγ signaling.

## Discussion

Achieving long term event free survival, overall survival, and decreasing disease reoccurrence for children with aggressive tumors who have undergone one of the various treatment options, have been clinically challenging. Neuroblastoma treatment regimens include observation, chemotherapy, surgery, radiation, and stem cell/bone marrow transplant depending on neuroblastoma stage. Consolidation therapies like differentiation therapy, antibody therapy, and immunotherapy are prescribed for treating minimal residual disease (Matthay et al., 1999; Yu et al., 2010; Lopci, 2015). Neuroblastoma tumors are often histopathologically diverse, displaying various degrees of cellular and extracellular maturation due to their neuroectodermal origin. Differentiation block is thus a characteristic of malignant neuroblastoma tumors, giving rise to tumor variants.

Naturally occurring retinoic acid isomers, ATRA and 9-cis retinoic acid (9-cis-RA) are key ligands for members of nuclear hormone superfamily which regulate retinoic acid mediated signaling (Heyman et al., 1992). The receptors (RXRs and RARs) mediate retinoid signal either by binding to specific DNA sequences like retinoic acid response elements (RAREs) as RAR-RXR heterodimers or RXR homodimers (Benkoussa et al., 2002; Idres et al., 2002). RXRs also heterodimerize with members of other nuclear hormone receptor family like PPAR, TH, VDR, LXRs, FXRs, PXRs, CARs, and many other orphan receptors (Kliewer et al., 1992; Evans and Mangelsdorf, 2014). PPARγ and RXR together bind as a complex specifically to PPAR response elements (PPREs) to regulate target gene expression. Besides this, the dimer can also interact with, and recruit coactivators to induce expression of specific genes (Forman et al., 1996; Ahmadian et al., 2013). Retinoid resistance is commonly observed *in vitro* as well as clinically, owing to the mutations in RAR gene or RAR receptor being truncated at C terminal end, and for many unknown reasons (Pemrick et al., 1994). Since its discovery, CDDO is known to be a multifunctional molecule exhibiting several pharmacological activities. Previous findings have also indicated the role of PPARγ stimulation in the differentiation of neuronal cell lines (Han S. et al., 2001; Jung et al., 2003; Chiang et al., 2014). CDDO has previously been through clinical trials for patients with relapsed and refractory leukemias; however, no responses were reported. CDDO also failed a tumor trial as it did not exhibit anti-tumor activity (Speranza et al., 2012).

In our study, we used IMR32 cell line which is a well-established *in vitro* model for experimenting differentiation using neurotrophic factors and synthetic compounds (Tumilowicz et al., 1970). IMR32 possesses most of the molecular signatures of aggressive neuroblastomas with unfavorable biological outcomes. Here, we report that simultaneous treatment with CDDO and ATRA induces neurite outgrowth, increases the percentage of cells bearing more than two neurites, reduces cell viability, up-regulates differentiation markers, and down-regulates *MYCN* mRNA level in IMR32 cells. We observed that maximum population of cells treated with CDDO especially in combination with ATRA underwent differentiation and eventually died. We noticed less reduction in viability with ATRA up to 5 days compared to the CDDO's individual and combination treatment. This was not unfamiliar as it is known that 9-cis-RA and 13-cis-RA are more potent anti-proliferative in nature (Han et al., 1995; Celay et al., 2013). Repeated treatments with CDDO after 5 days were found cytotoxic to cells, whereas, in combination with ATRA, gradual loss of cells bearing neurites and decrease in viability was observed. Treatment with ATRA at 15 μM concentration for 5 days induced morphological differentiation as evidenced by neurite outgrowth without affecting growth in IMR32 cells (data not shown). The morphological changes between the groups were quite distinctive. Following CDDO and combination treatment, the extending neurites were found to make contacts with other neurites and cell bodies of neighboring cells, forming a branched neurite network. On the contrary, less neurite network was found in ATRA treated cells up to 5 days. Non-linear correlation between mRNA levels and protein abundance of differentiation markers (NSE and β3-tubulin) may be due to differential post-transcriptional regulation exerted by CDDO and ATRA. Additionally, as observed in our study, the differentiation markers, although not regulated at transcriptional levels by ATRA, were regulated at translational levels, evidenced by immunofluorescence studies. Overexpression of Aryl Hydrocarbon Receptor (AHR) in SK-N-DZ cell line demonstrated neurite outgrowth without up-regulation of NSE mRNA levels (Wu et al., 2014). In another study, NSE mRNA up-regulation to 1.5-fold sufficiently displayed neurite formation in SHSY5Y cells treated with 10 μM retinoic acid. Also the NSE mRNA level in IMR32 cells was found to be decreasing on day 5 (Maresca et al., 2012). In our study, in spite of distinct immunolabelling of β3-tubulin observed in differentiated cells, its mRNA levels were not found to be regulated possibly due to a post-transcriptional auto-regulatory mechanism. Co-translational recognition of an emerging tetra-peptide (MREI) targets the ribosome bound tubulin mRNA for selective degradation. This process affects the cytoplasmic tubulin mRNAs and is dependent on the levels of un-polymerized tubulin subunit levels (Yen et al., 1988; Bachurski et al., 1994).

Because CDDO is a known partial PPARγ agonist, to further investigate if the morphological changes and reduced viability required active PPARγ signaling, we used T0070907, a selective PPARγ antagonist (Lee et al., 2002). Pretreatment with T0070907 significantly reduced neurite lengths and increased viability in IMR32 cells implying that these effects of CDDO were dependent on PPARγ receptor mediated signaling. Transcriptional activation of PPRE by CDDO has been shown in several cell lines using reporter assays (Lapillonne et al., 2003; Mix et al., 2004; Chintharlapalli et al., 2005). Similarly, synthetic PPARγ agonists have been shown to inhibit cell growth in several neuroblastoma cell lines (Valentiner et al., 2005). Our immunofluorescence analysis shows that T0070907 considerably decreased the expression of the differentiation markers β3-tubulin and NSE, suggesting that active PPARγ signaling was in part accountable for CDDO induced differentiation in IMR32 cells. The enhanced differentiation in IMR32 cells, by the combination of ATRA and CDDO, could perhaps be due to simultaneous activation of genes regulated under RAREs and PPREs; which mutually initiates a crosstalk between signaling pathways, influencing cell growth and differentiation. In the combination treatment, a decrease in cell viability accompanied the differentiation process, which was not observed in ATRA treated cells.

Restricted to embryonic development, MYCN proto-oncogene is a transcription factor belonging to basic helix loop helix zipper class, which governs assorted fundamental cellular processes. MYCN amplification is central to many advanced stage neuroblastomas which exhibit poor prognosis. MYCN shares the burden of being the cause of neuroblastoma by preventing differentiation and maintaining pluripotency at the expense of cells future (Cotterman and Knoepfler, 2009; Varlakhanova et al., 2010). MYCN along with other transcriptional cofactors regulates gene expression by heterodimerizing with Max and binding to E box sites mainly CATGTG; however, in the case of MYCN amplification, this binding becomes ambiguous (Murphy et al., 2009). In our study, we observed 80% reduction in *MYCN* mRNA level in CDDO treated cells whereas ATRA did not regulate *MYCN* mRNA level up to day 5. This would explain ATRA's inability to inhibit proliferation compared to CDDO as *MYCN* down-regulation is associated with and required for growth inhibitory responses of cells (Schweigerer et al., 1990; Negroni et al., 1991). *MYCN* down-regulation by CDDO resulted in re-expression of NDRG1 which is otherwise known to be repressed in neuroblastoma (Li and Kretzner, 2003). A study on *NDRG1* expression in tumors obtained from neuroblastoma patients revealed its clinical significance with less expression being associated with tumor progression and poor survival (Matsushita et al., 2013). In our study, ATRA treated cells failed to up-regulate NDRG1. A simultaneous observation was that the differentiation induced by CDDO alone and in combination with ATRA was accompanied by accumulation of lipid droplets by day 5 (Supplementary Figure 11). This effect could supposedly be an aftermath of *MYCN* reduction or other cellular events (Gulaya et al., 1989; Tanaka et al., 2012; Zirath et al., 2013; Muller et al., 2014).

Similar to MYCN, ALK amplification, and gain of function mutations are often correlated with poor outcome for neuroblastomas. IMR32 cells contain wild type ALK and co-amplification of MYCN and ALK as fused single amplicon is also observed (Fransson et al., 2015). In a study performed by Passoni et al. using siRNA and small kinase inhibitor to inhibit active ALK in IMR32 cells, demonstrated that wild type ALK is in part functionally responsible for the oncogenic property of IMR32 cells. Stimulation with the anti-ALK antibody, mAb46 showed a 40% increase of *MYCN* mRNA in IMR32 cells validating that active ALK contributes to *MYCN* level (Passoni et al., 2009). ATRA has been previously shown to down-regulate ALK in neuroblastoma cell lines and induce apoptosis selectively in those harboring activated ALK (NB-39-nu and SH-SY5Y) (Futami and Sakai, 2010). Our finding indicates that ATRA, but not CDDO, significantly decreases ALK expression.

Additionally, ATRA was found to increase PPARG level. CDDO did not up-regulate the basal level of PPARγ in IMR32 cells, however previous findings in human SW-1353 chondrosarcoma cells report an increase in PPRE activity without change in mRNA and protein expression (Mix et al., 2004). Perhaps CDDO and ATRA together are able to regulate a certain subset of genes causing cells to differentiate and die eventually, a phenomenon not observed in ATRA treated cells up to 5 days. Moreover, CDDO as a partial PPARγ agonist may recruit certain co-activators and release co-repressors regulating transcription of tissue specific genes involved in differentiation and cell survival. It has previously been shown using LAN5 cells that GW1929, a synthetic PPARγ agonist can induce neurite outgrowth, cease proliferation and reduce MYCN expression. PPARγ expression is also found to correlate with maturation stage of primary neuroblastoma tissue with differentiated phenotype having high nuclear PPARγ expression (Han S. W. et al., 2001). Contrarily, N2 media induced SHSY5Y differentiation and displayed cytoplasmic accumulation of PPARγ. Moreover, silencing PPARγ improved differentiation associated with up-regulation of other two PPAR isoforms (PPARβ/δ and PPARα) (Di Giacomo et al., 2017). Besides, the phosphorylation status of PPARγ also decides the responsiveness to PPARγ agonists. Rosiglitazone induced PPRE activity in SK-N-AS but not in SH-SY5Y, probably due to presence of the phosphorylated PPARγ in SHSY5Y cells (Cellai et al., 2006). Thus, the sensitivity of different neuroblastoma cell lines to different PPARγ agonist varies owing to their cellular heterogeneity (Servidei et al., 2004). Furthermore, CDDO being electrophilic in nature can interact with several proteins affecting their function. Using affinity purification and mass spectroscopic approach, 577 binding partners were identified for a CDDO-Im, an imidazolide derivative of CDDO (Yore et al., 2011).

CDDO and ATRA did not exhibit additive or synergistic activation of non-genomic mechanisms like ERK and CREB activation. Despite the combination being more effective in neurite outgrowth and growth inhibition, the downstream classic effectors like ERK and CREB were not found active. This suggests that CDDO and ATRA activated different signaling pathways, contributing to the biological effects observed. In IMR32 cells, we observed an increase in ERK and CREB phosphorylation in ATRA treated cells compared to control cells. Retinoic acid treatment in SK-N-SH and BE(2)-C cell lines have been earlier reported to promote cell survival during the differentiation process, suggesting a potential mechanism for neuroblastoma resistance to retinoic acid therapy (Qiao et al., 2012). In our study, CREB activation in IMR32 cells by ATRA possibly leads to transcription of its target genes, initiating pro-survival signaling pathways (Bonni et al., 1995). In fact, CDDO considerably reduced ATRA induced ERK and CREB phosphorylation in the combination treatment, implying that cells die as/after they are differentiated. Thus, CDDO appears to induce differentiation and cell death in IMR32 cells via CREB and ERK independent mechanisms. However, antagonizing PPARγ receptor caused increased CREB and decreased ERK activation in CDDO treated cells, suggesting that PPARγ receptor signaling was responsible for the observed effects of CDDO. Therefore, CDDO being a synthetic and ATRA being a physiological molecule seem to trigger different molecular pathways leading to a common effect i.e., differentiation in IMR32 cells. Altogether, it appears that CDDO in combination with ATRA induces IMR32 differentiation via CREB independent and PPARγ dependent signaling mechanisms.

Differentiation therapy aims to reprogram cells instead of directly killing them and thus it is received as a treatment option that is mild on young children. Our concern was to focus on evaluating CDDO from a perspective of differentiation therapy instead of chemotherapy, which is known to have various side effects. Because retinoic acid is in clinical practice, we attempted testing the effect of CDDO in combination with ATRA on neuroblastoma differentiation. In conclusion, our findings provide early evidence that combination treatment would be a more effective choice than a single drug treatment in differentiating and killing aggressive cells like IMR32. Moreover, differentiated tumors are associated with favorable outcome. Additionally, many neuroblastomas are found refractive to retinoic acid therapy *in vivo* and therefore combination therapy holds a chance in the treatment of neuroblastoma. The results contribute to the current knowledge on neuroblastoma differentiation by showing that CDDO induced neurite outgrowth, decreased viability, up-regulation of differentiation markers and down-regulation of active CREB were PPARγ dependent events in IMR32 cells. The highlight of this research in our opinion is that CDDO and ATRA together are capable of triggering events that help the IMR32 cells to differentiate and die. The *in vivo* efficacy of this combination in neuroblastoma xenograft models is yet to be studied.

## Author contributions

PR conceptualized and designed the work. NC performed the experiments. PR and NC wrote the manuscript. PT and CL contributed in the discussion.

### Conflict of interest statement

The authors declare that the research was conducted in the absence of any commercial or financial relationships that could be construed as a potential conflict of interest.
