# Supplementary material for: CDDO and ATRA Instigate Differentiation of IMR32 Human Neuroblastoma Cells
Source: Front Mol Neurosci. 2017 Sep 26;10:310. doi: 10.3389/fnmol.2017.00310 (PMC5623017; doi:10.3389/fnmol.2017.00310)

Supplementary Material

CDDO and ATRA instigate differentiation of IMR32 human neuroblastoma cells

Namrata Chaudhari[1], Priti Talwar[1], Christian Lefebvre d'Hellencourt[2], Palaniyandi Ravanan[1]*

[1] Apoptosis and Cell Survival Research Lab, Department of Biosciences, School of Biosciences and Technology, VIT University, Vellore, India.

[2] Université de La Réunion, INSERM, UMR 1188 Diabète athérothombose Thérapies Réunion Océan Indien (DéTROI), Saint-Denis de La Réunion, France

* Correspondence: Dr. P. Ravanan: ravanan.p@vit.ac.in

Supplementary Figure 1: Neurite traced images in triplicates for (A) Vehicle control; (B) CDDO 0.7 µM; (C) CDDO 0.7 µM + ATRA 10 µM; (D) ATRA 10 µM; (E) T0070907 5 µM + CDDO 0.7 µM; (F) T0070907 5 µM; (G) Hemocytometer was used to set scale to convert pixels to µM.

A

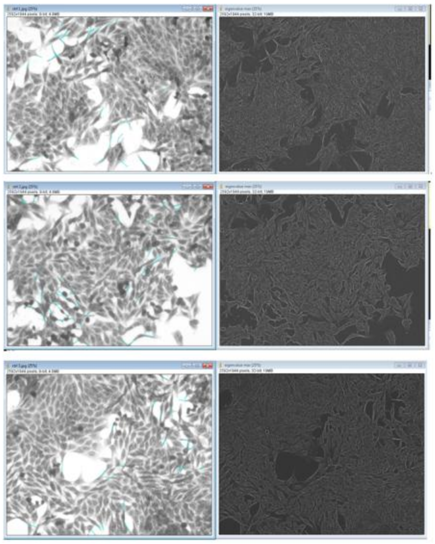

B

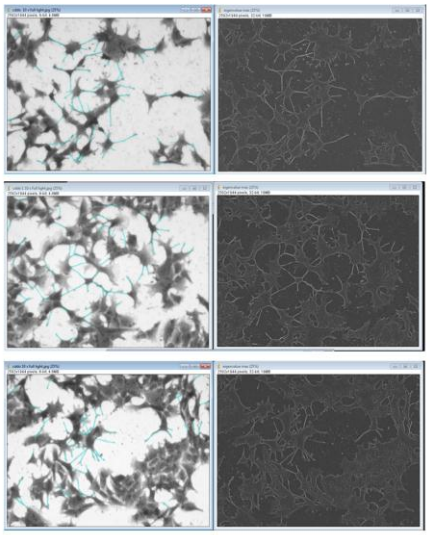

C

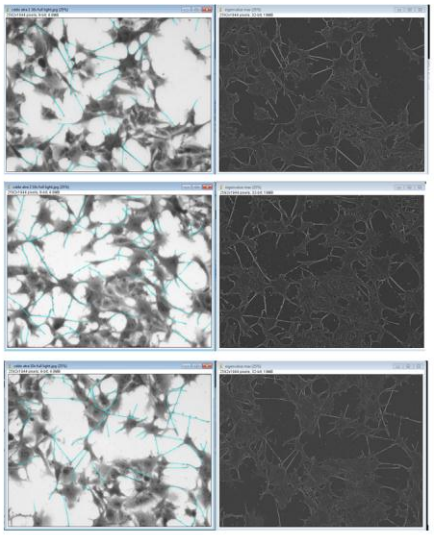

D

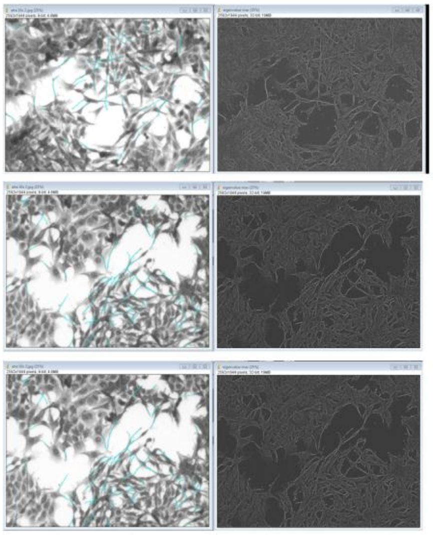

E

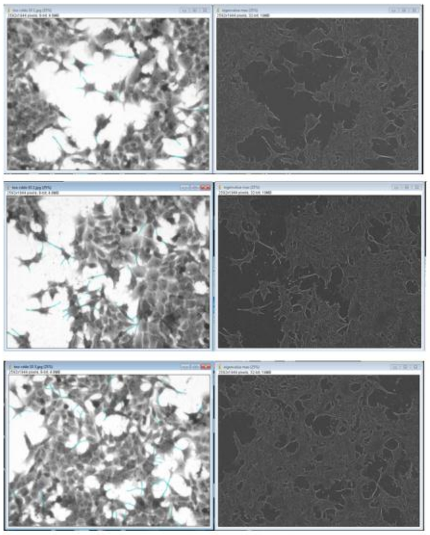

F

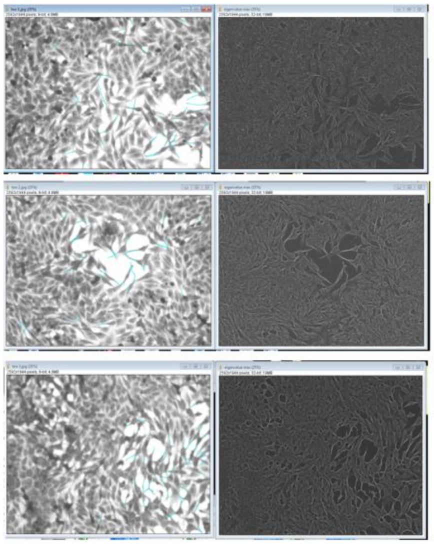

G

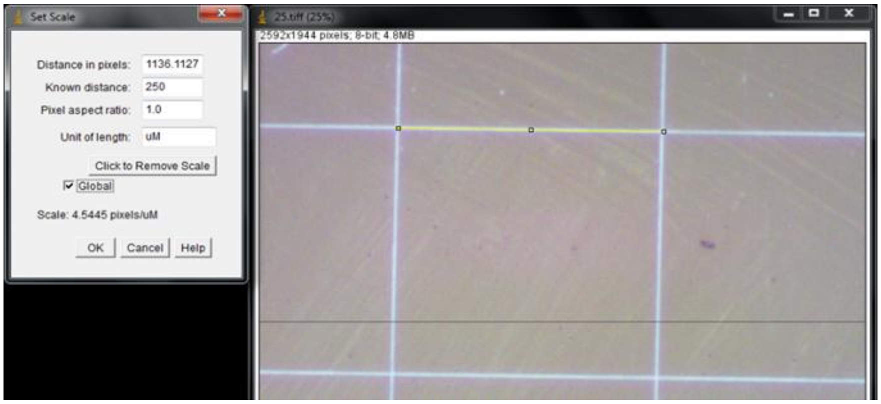
Set Scale
Distance in pixels: 1136.1127
Known distance: 250
Pixel aspect ratio: 1.0
Unit of length: uM
Click to Remove Scale
Global
Scale: 4.5445 pixels/uM
OK
Cancel
Help
25.tiff (25%)
2592x1944 pixels; 8-bit; 4.8MB

Supplementary Figure 2: CDDO and ATRA together enhance neurite outgrowth while antagonist inhibits CDDO induced neurite outgrowth: (A) Neurite tracings were performed for 10 images per treatment condition to demonstrate concentration dependent increase in neurite length following CDDO treatment. ATRA and CDDO in combination display increased neurite lengths. (B) Neurite tracings were performed for 10 images per treatment condition to demonstrate the inhibitory effect of PPARγ antagonist T0070907 (T00) 5 µM on CDDO induced neurite lengths.

A

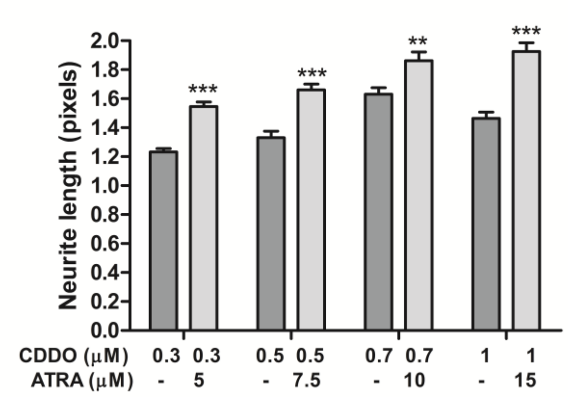

B

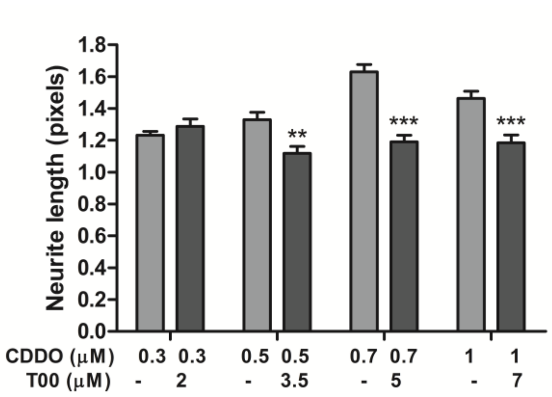

Supplementary Figure 3: Cells were treated with CDDO and ATRA alone and in combination and assessed for viability and morphological changes. (A) Trypan blue dye exclusion method was used to study the viability for various time points up to 8 days. '*' denotes significance with respect to control; '@' denotes significance with respect to CDDO; '#' denotes significance with respect to ATRA and '!' denotes significance with respect to CDDO + ATRA. (B) Phase contrast images reflect the morphological changes occurring over a period of 10 days. The control and ATRA treated cells reached confluency by day 10. As the monolayer started rolling over from the edges (small figure) viability was not estimated for day 10; scale bar 100 µM.

A

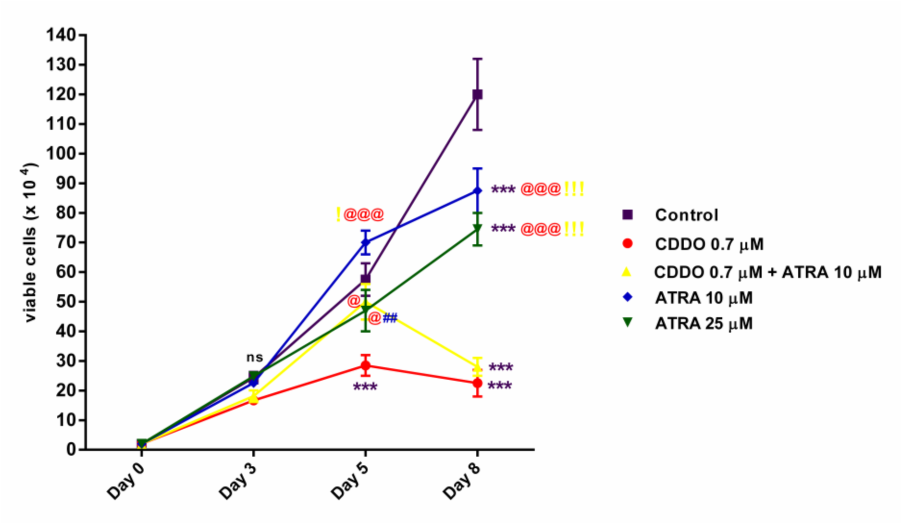

B

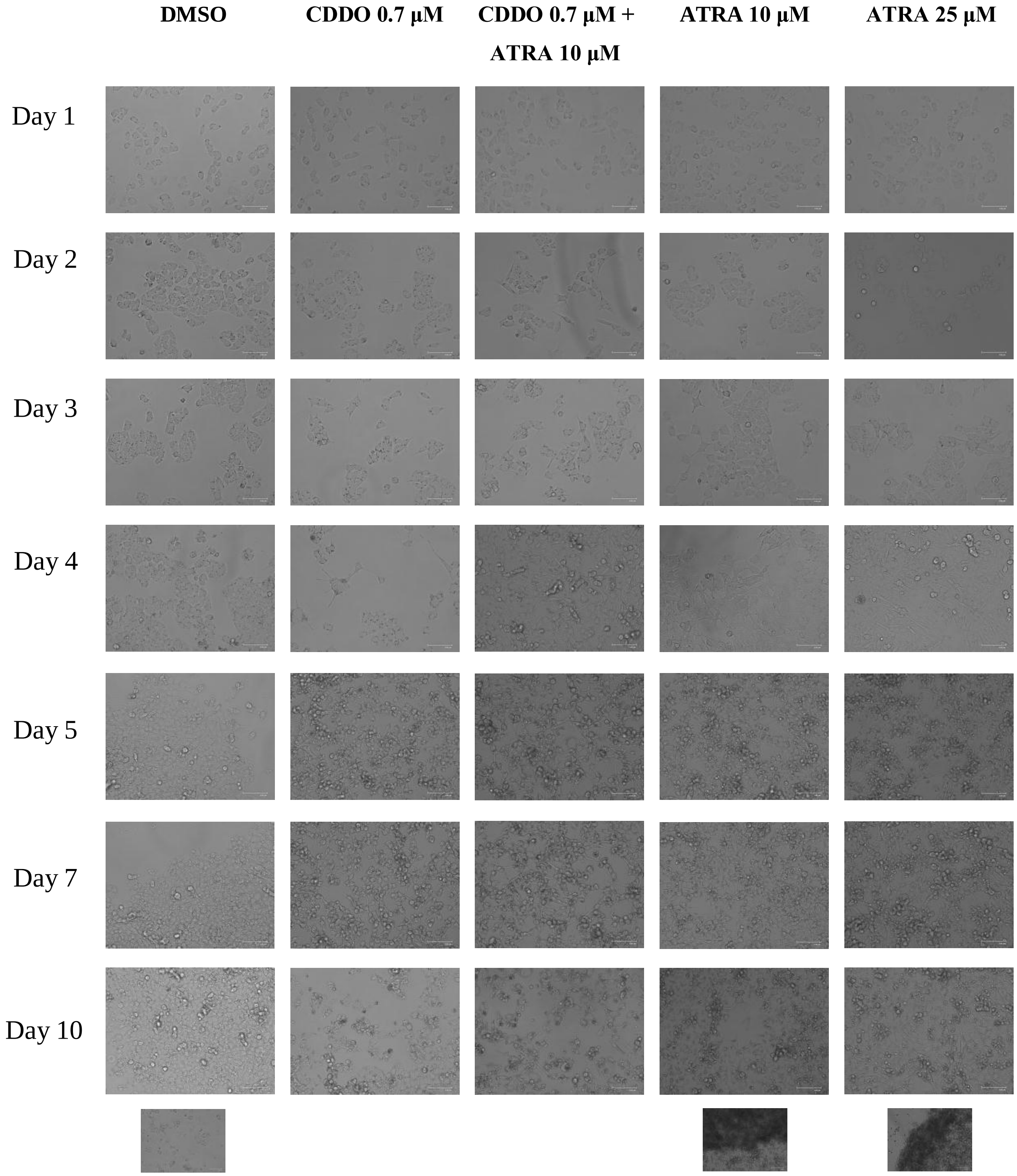
DMSO
CDDO 0.7 μM
CDDO 0.7 μM + ATRA 10 μM
ATRA 10 μM
ATRA 25 μM
Day 1
Day 2
Day 3
Day 4
Day 5
Day 7
Day 10

Supplementary Figure 4: CDDO induced neurite outgrowth in Neuro2a cells: Neuro2a cells were treated with CDDO 1 µM (A, B); 1.5 µM (C, D), 2 µM (E, F), ATRA 10 µM (G, H); vehicle control (I, J) and imaged at higher (A, C, E, G, I scale bar 50 µM) and lower (B, D, F, H, J scale bar 100 µM) magnification after methylene blue staining at day 5.

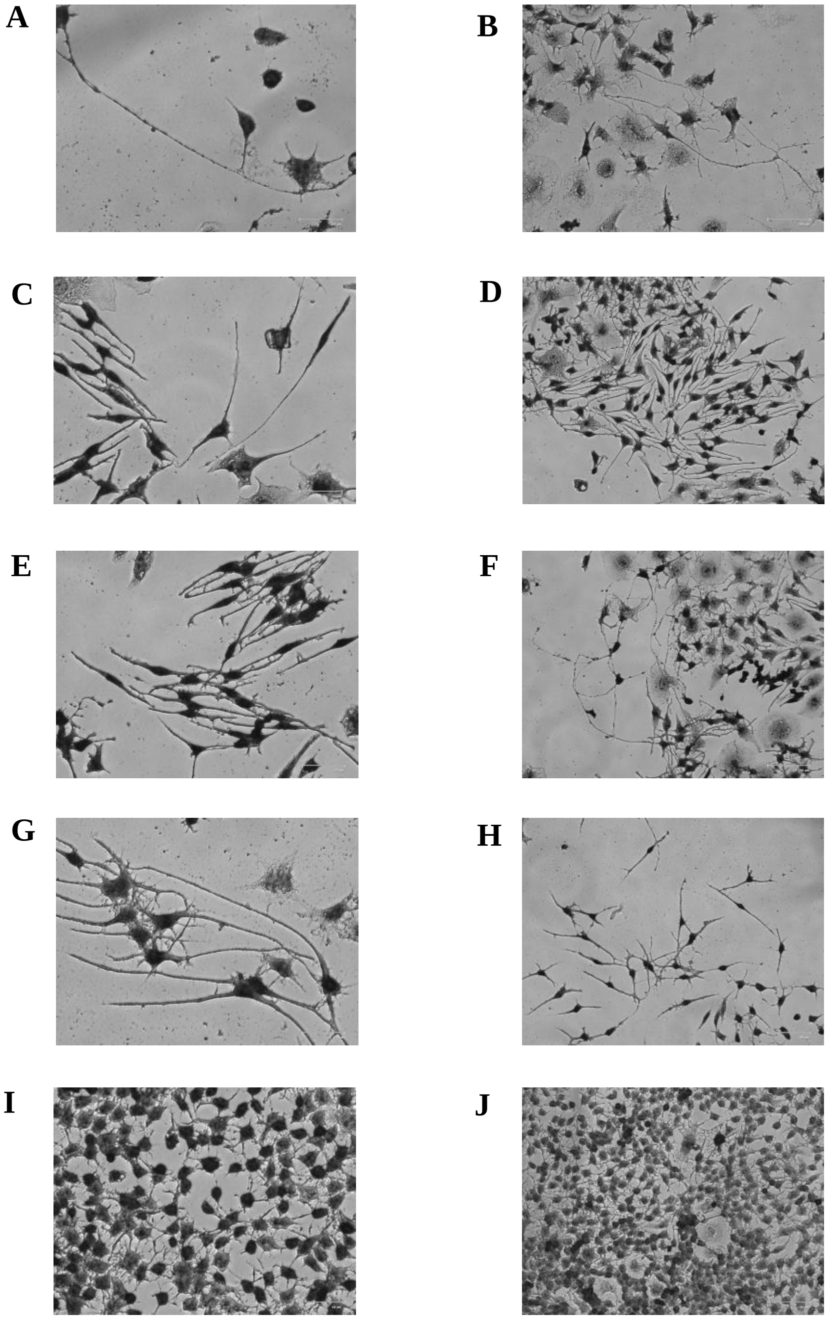

Supplementary Figure 5: CDDO failed to induce neurite outgrowth in SHSY5Y cells: SHSY5Y cells were treated with CDDO 0.7 μM (A, B); CDDO 0.7 μM + ATRA 10 μM (C, D); ATRA 10 μM (E, F) and vehicle control (G, H) and imaged at higher (A, C, E, G scale bar 50 μM) and lower (B, D, F, H scale bar 100 μM) magnification after methylene blue staining at day 5.

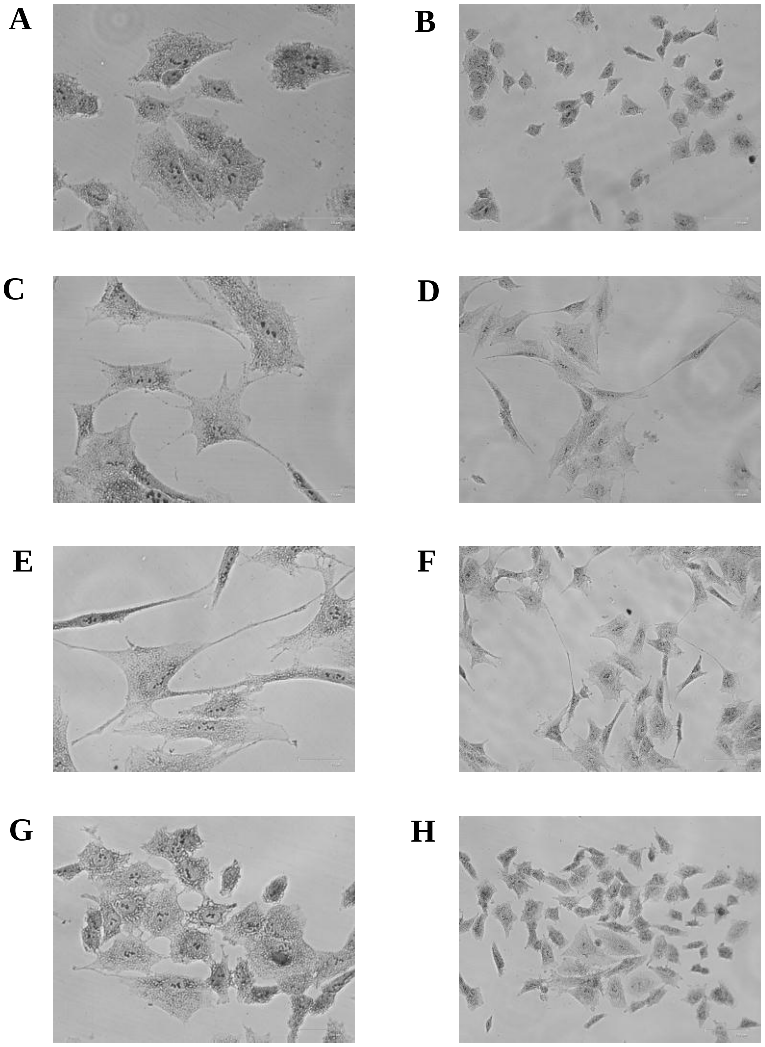

Supplementary Figure 6: IMR32 cells were treated with CDDO and ATRA in serum free condition. Treated and untreated cells exhibited unhealthy morphology within 48 h. Scale bar 100 µM

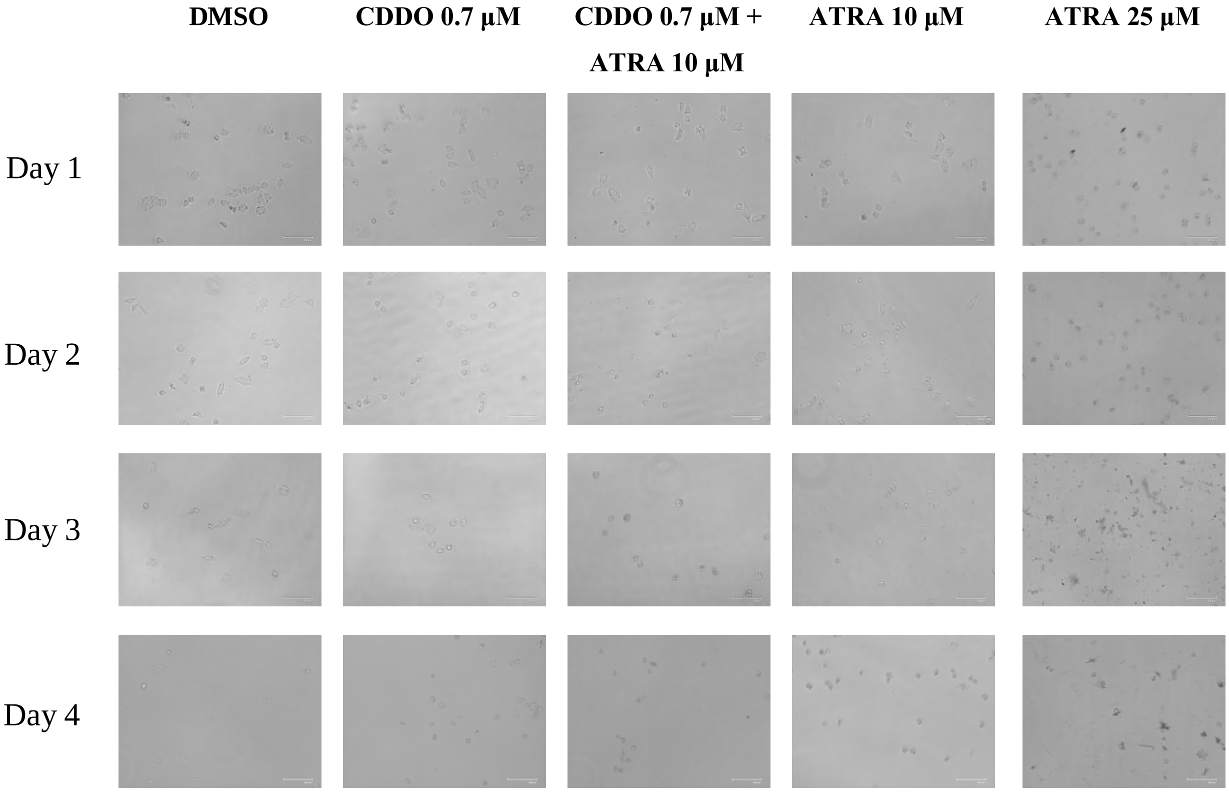

Supplementary Figure 7: IMR32 cells were treated with CDDO and ATRA in 0.5% serum containing media. Scale bar 100 µM

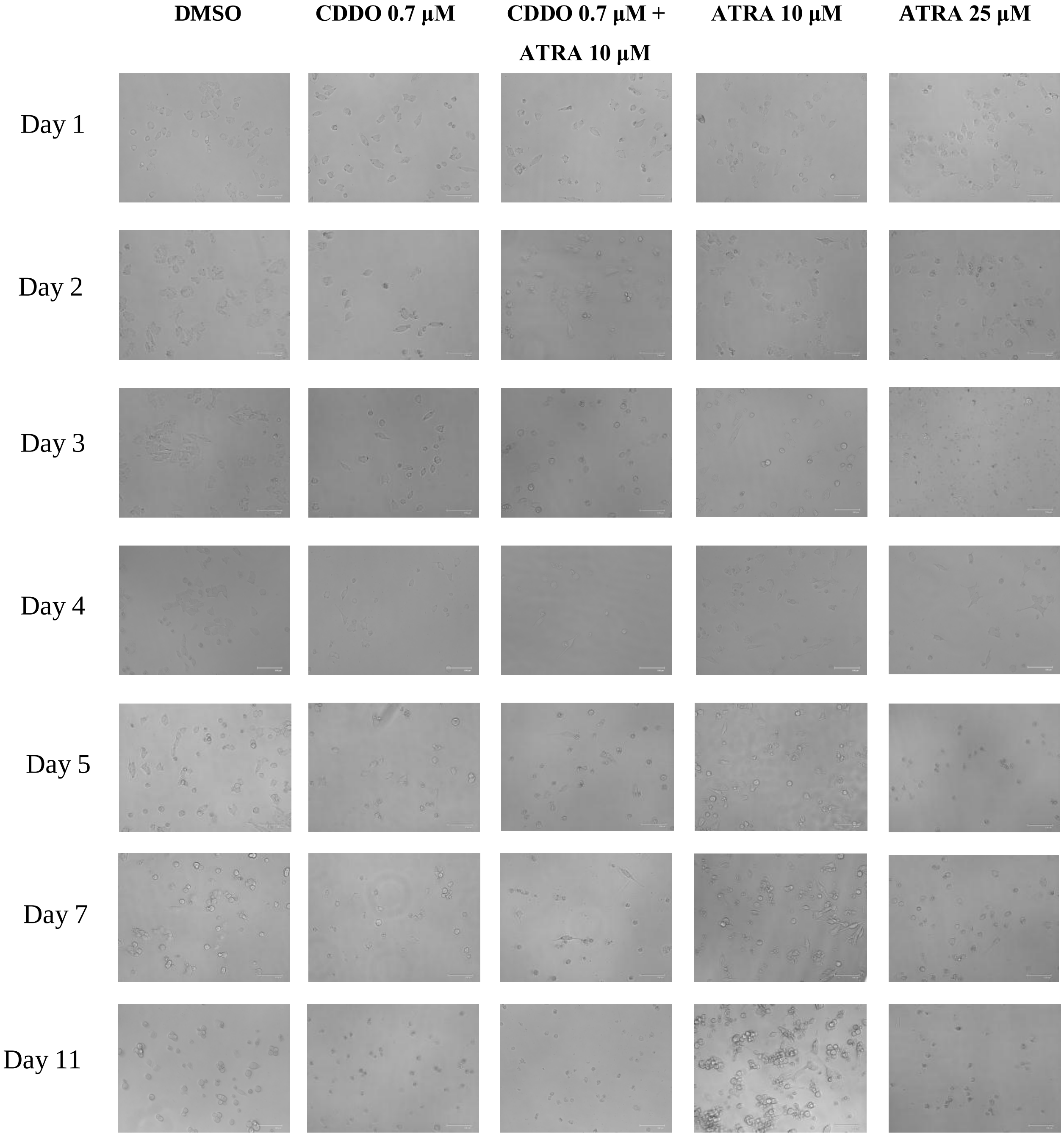

Supplementary Figure 8: Cellular localization of differentiation marker: Control and treated cells were immunostained with Tuj1 on day 5 and imaged. Scale bar 50 μM

A

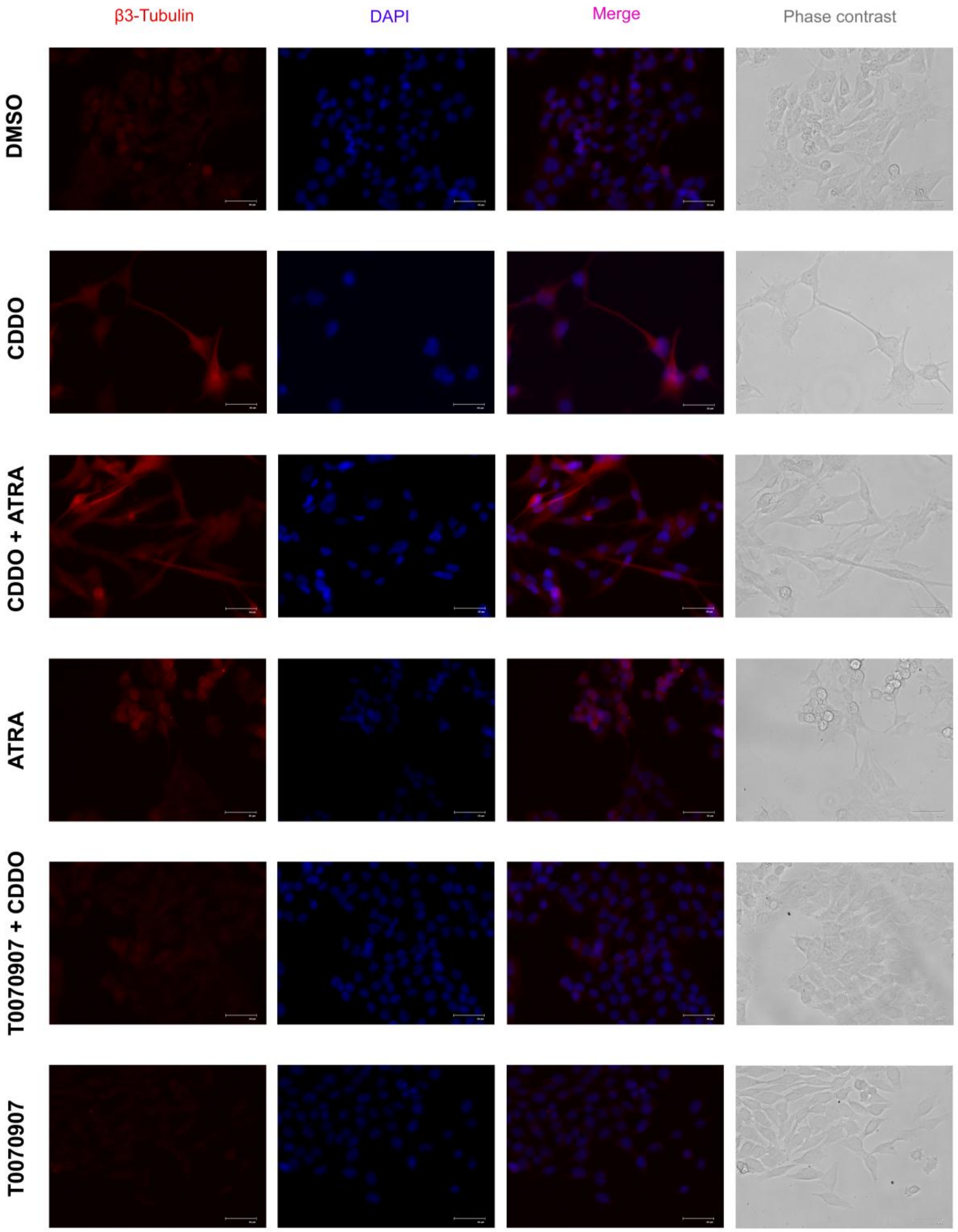

Supplementary Figure 9: Cellular localization of differentiation marker: Control and treated cells were immunostained with NSE on day 5 and imaged. Scale bar 50 μM

A

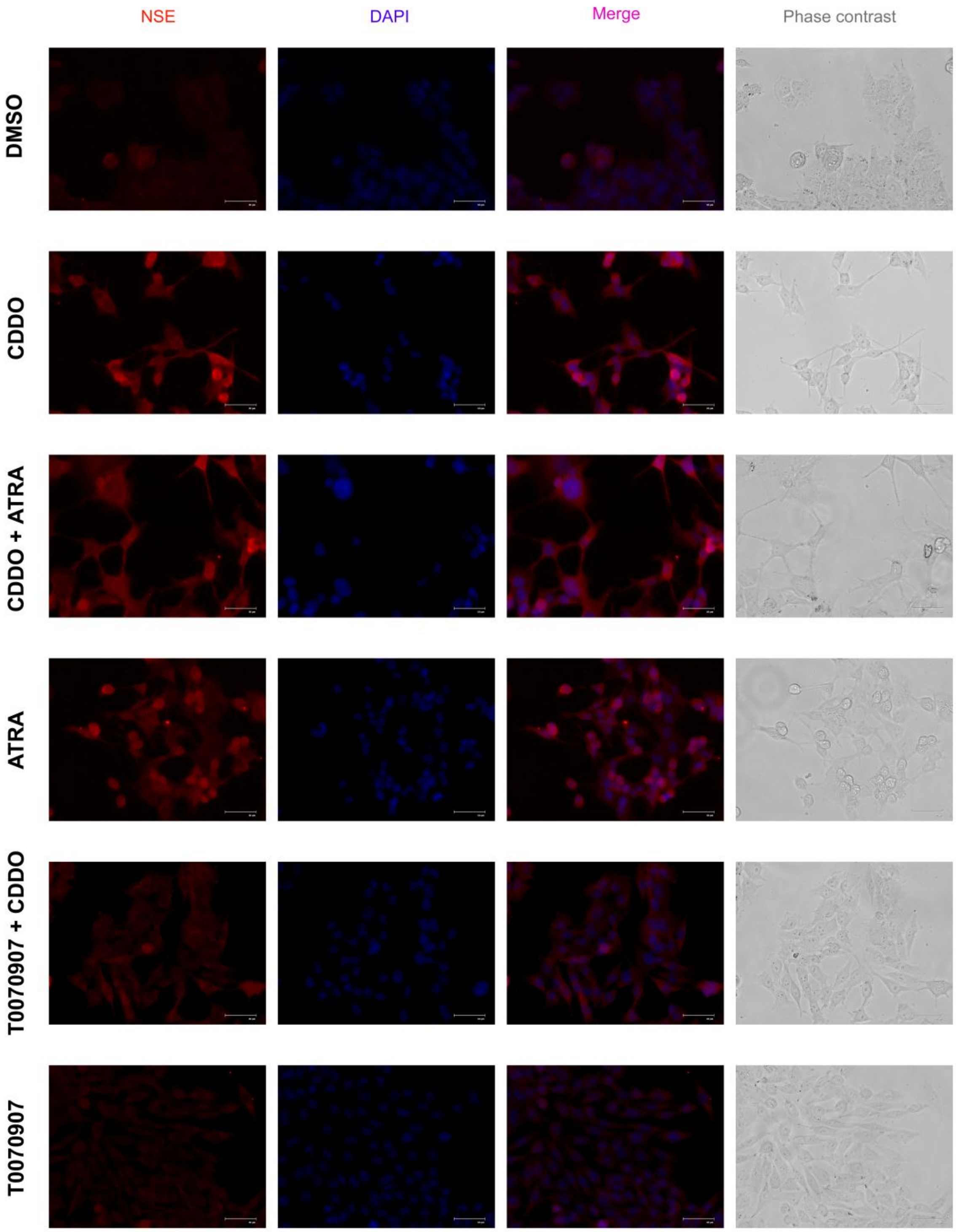

Supplementary Figure 10: Primary antibody control: Cells were cultured on coverslips, fixed, and incubated with primary antibody dilution buffer. Alexa Fluor 594 was used as secondary antibody and DAPI to stain the nucleus. Scale bar 50 μM

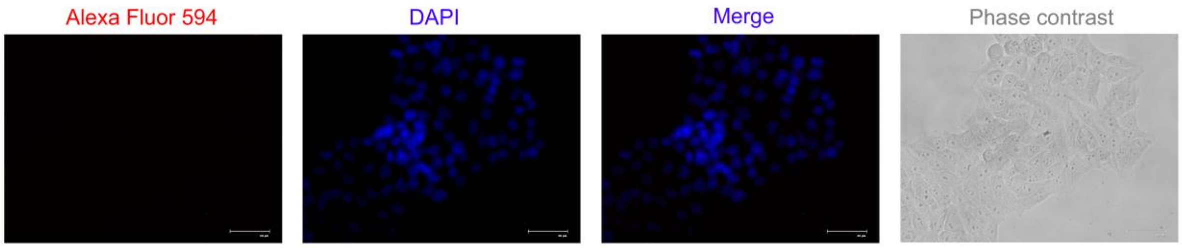

Supplementary Figure 11: Lipid accumulated in response to CDDO and ATRA treatment. IMR32 cells were treated with CDDO 0.7 μM (A); ATRA 10 μM (C); CDDO 0.7 μM + ATRA 10 μM (B); vehicle control (D) and imaged after oil red O staining. Magnification 200x.

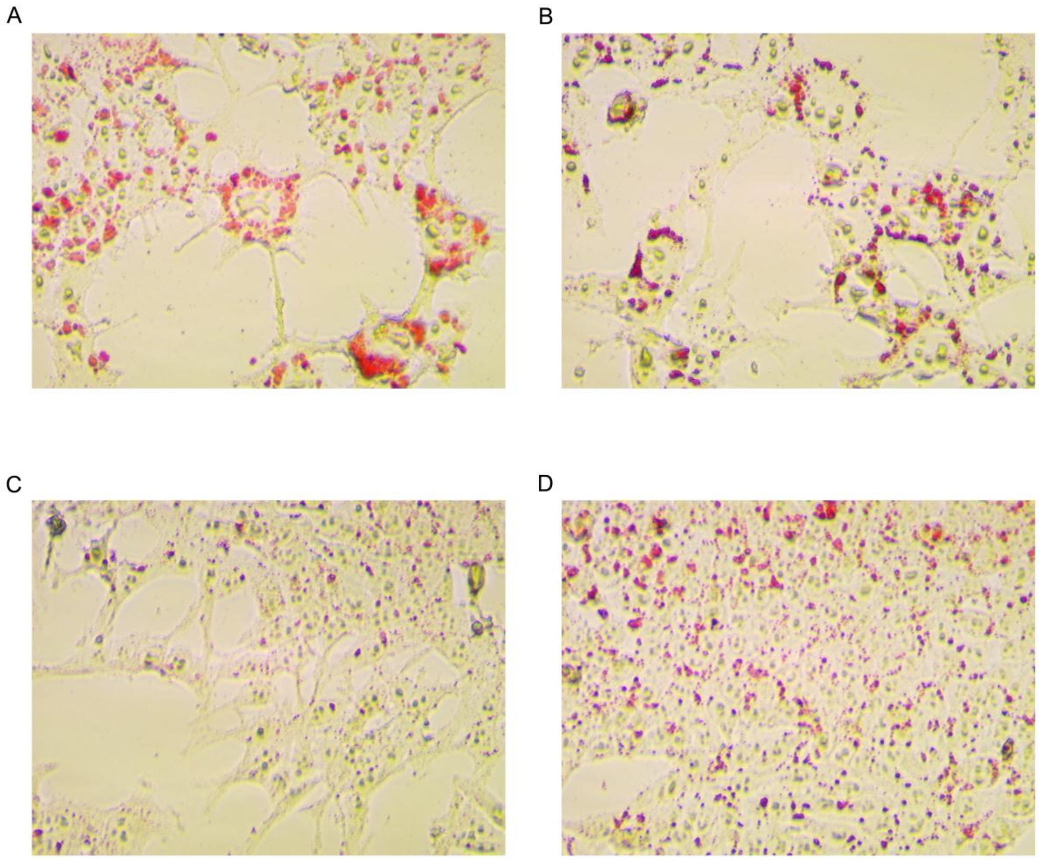

Supplement: Supplementary file 1 [file DataSheet1.pdf]
